# The clinical outcomes of xenografts in the treatment of burn patients: a systematic review and meta-analysis

**DOI:** 10.1186/s40001-023-01505-9

**Published:** 2023-11-16

**Authors:** Rana Irilouzadian, Amirmohammad Khalaji, Hediyeh Baghsheikhi, Roham Sarmadian, Soodabeh Hoveidamanesh, Tayyeb Ghadimi, Siamak Farokh Forghani

**Affiliations:** 1 Burn Research Center, Iran University of Medical Sciences, Tehran, Iran; 2 School of Medicine, Tehran University of Medical Sciences, Tehran, Iran; 3 School of Medicine, Shahid Beheshti University of Medical Sciences, Tehran, Iran; 4 Infectious Diseases Research Center, Arak University of Medical Sciences, Arak, Iran

**Keywords:** Burn, Xenografts, Animal transplant, Wound

## Abstract

**Background:**

Although autografts are not feasible in patients with extensive burn wounds, allografts and xenografts can be used for temporary coverage. In this systematic review and meta-analysis, we compared the outcomes of xenografts and the standard treatment of burn wounds.

**Methods:**

International online databases were searched for English articles comparing xenografts with routine treatment in the burn patients. The random-effects model was used to estimate standardized mean differences (SMD) or odds ratios (OR) with a 95% confidence interval (CI).

**Results:**

From a total of 7144 records, 14 studies were included in our review after screening by title and abstracts followed by full-texts. No significant difference in hospital stays was found between the mammalian xenografts and control groups (SMD [95% CI] = − 0.18 [− 0.54–0.18]). The mean number of dressing changes was significantly lower in both mammalian xenografts compared to the controls (SMD [95% CI] = − 1.01 [− 1.61–− 0.41]) and fish xenografts compared to controls (SMD [95% CI] = − 6.16 [− 7.65–− 4.66]). In the fish xenografts, re-epithelialization time was significantly lower compared to controls (SMD [95% CI] = − 1.18 [− 2.23–− 0.14]).

**Conclusions:**

Xenografts showed a significantly lower number of dressing changes and fish xenografts showed significant benefit in re-epithelialization compared to routine treatment. The beneficial results of xenografts suggest further research in the use of different types of xenografts in patients with extensive burn.

**Supplementary Information:**

The online version contains supplementary material available at 10.1186/s40001-023-01505-9.

## Introduction

Burn injuries result in inflammation and metabolic disturbances, leading to shock, multi-organ failure, and considerable morbidity and mortality. Burn injuries are caused by exposure to thermal, chemical, and electrical sources and radiation leading to tissue damage by different mechanisms. Moreover, the management and outcome of burn injuries greatly result from the depth and size of the wound [1].

Deep burns are more likely to have complications, such as wound infection, sepsis, shock, and scarring tissue contraction [2]. Wound infections and prolonged healing time increase the risk of scars [3]. Therefore, prompt coverage of the burn wounds and necessary treatment based on the depth and size of the wound is critical. Suggested treatments are topical silver agents, biological dressings, including amniotic membrane, allografts, xenografts, bioengineered dressings, enzymatic debridement, and surgery [4].

Silver sulfadiazine (SSD) is an antimicrobial topical agent with a low risk of bacterial resistance, adverse effects, and toxicity. It has been used for treating chronic and burn wounds for a long time but the abundant number of dressing changes and the resultant pain propose the necessity of better treatment [5].

The gold standard treatment of deep partial thickness and full thickness burn is early excision and skin grafts [6, 7]. Contrarily, patients with extensive burn wounds do not have enough available donor sites for autografts; therefore, temporary coverage with allografts, xenografts, and skin substitutes is used [8].

Biological skin substitutes must have adequate strength, flexibility, adhesion to the wound, good aesthetic results, and remodeling ability to provide an optimal wound repair and healing. Moreover, their safety in terms of risk of infectious disease transmission, microorganism penetration, toxicity, oncogenicity, and allergenicity is concerning [9]. A Porcine graft is a good candidate for burn wound dressing used as they act as a barrier for microorganism entrance and loss of heat and fluid. Moreover, studies have shown that porcine grafts have decreased the pain and required fewer dressing changes [7, 10–12].

Another commonly used xenograft is derived from fish which has been reported in some cases of burn wound treatment and neovaginoplasty [13, 14]. Nile Tilapia fish skin has demonstrated leather-like resistance, similar to human skin, noninfectious microbiota, and favorable results in animal models with burn wounds [15, 16]. Many trials have compared outcomes between xenografts and other common treatments in burns. Hence, in this systematic review and meta-analysis, we compared outcomes (e.g., re-epithelialization time, number of dressing changes, and hospital stay) between xenografts with other treatments of burn patients.

## Methods

This systematic review and meta-analysis were designed and performed in accordance with “Preferred Reporting Items for Systematic Reviews and Meta-Analyses” (PRISMA) [17]. Registration of the protocol is made on The International Prospective Register of Systematic Reviews (PROSPERO) (https://www.crd.york.ac.uk/prospero/display_record.php?ID=CRD42022373748).

### Eligibility criteria

Human or animal in vivo investigation of the effects of xenografts in burn wounds was determined as the fundamental inclusion criterion.

(P) Population: Burn patients.

(I) Intervention: Xenograft/Animal transplant/Animal graft.

(C) Comparison: Open.

(O) Outcomes: Open.

(S) Study: Clinical trials and animal studies.

According to these PICOS questions, we designed the following clinical question: in burn patients, what are the outcomes of the use of xenografts compared to routine treatment? Exclusion criteria included studies before the year 2000, studies other than trials and animal studies, and the studies that used genetically modified or not skin xenografts. Articles without full-text or not in English were excluded as well.

## Search strategy

We systematically searched PubMed, Cochrane Library, Scopus, and Web of Science without any filters or limitations until October 28, 2022. Keywords were Xeno* AND burn* with other related search terms shown in Additional file 1: Table S1. Duplicates were removed after the search.

## Study selection

Two independent reviewers (R.S. and H.B.) carried out the selection of studies. After removing the duplicates and screening the articles based on their title and abstracts, full-text of studies were retrieved to select the relevant studies according to the inclusion and exclusion criteria. Any disagreement during the article selection was resolved thanks to one independent investigator (R.I.).

## Data extraction

The data extraction process was performed by two independent researchers (H.B. and R.I.). The extraction tables included the name of the authors, country of origin, year of publication, study design, participant characteristics, including number, age, and TBSA, type of xenograft, detailed information about the surgeries that were operated on intervention and control groups, outcomes and complications in both groups. In addition, the review authors looked for the sources of funding for the studies included in the review.

## Risk of bias assessment

Two independent reviewers (H.B. and R.I.) evaluated the risk of bias in each article by means of "Cochrane Handbook for systematic reviews of interventions, version 5.1.0" [18]. We evaluated as low, some concerns, or high risk of bias the following six quality criteria: random sequence generation, allocation concealment, patient blinding, outcome blinding, incomplete outcome data, and selective reporting. Finally, a third independent reviewer (S.H.) resolved any disagreement during this step. The Cochrane tool of risk of bias assessment (RoB) was used to evaluate the quality of randomized studies [18]. The Risk Of Bias In Non-randomized Studies – of Interventions (ROBINS-I) [19] was used to assess the quality of the non-randomized studies and the SYRCLE tool was used for the animal studies [20].

## Statistical analysis

Standard mean difference (SMD) with 95% confidence intervals (CI) was used for comparing xenografts with other treatments of burns. Pairwise meta-analyses were conducted using RevMan software (Review Manager Version 5.3; The Cochrane Collaboration, Copenhagen, Denmark). Statistical significance was defined as *P* < 0.05 for all analyses. Forest plots were (JUSTIFICACIÓN DEL TEXTO) created to illustrate the effects in the meta-analysis of the global estimation. The heterogeneity of ≤ 25% was considered as low, 26–75% as moderate, and > 75% as high [21]. Due to high heterogeneity, the random-effect model (DerSimonian and Laird) was used for the meta-analyses.

## Results

### Literature search results

**Fig. 1:**
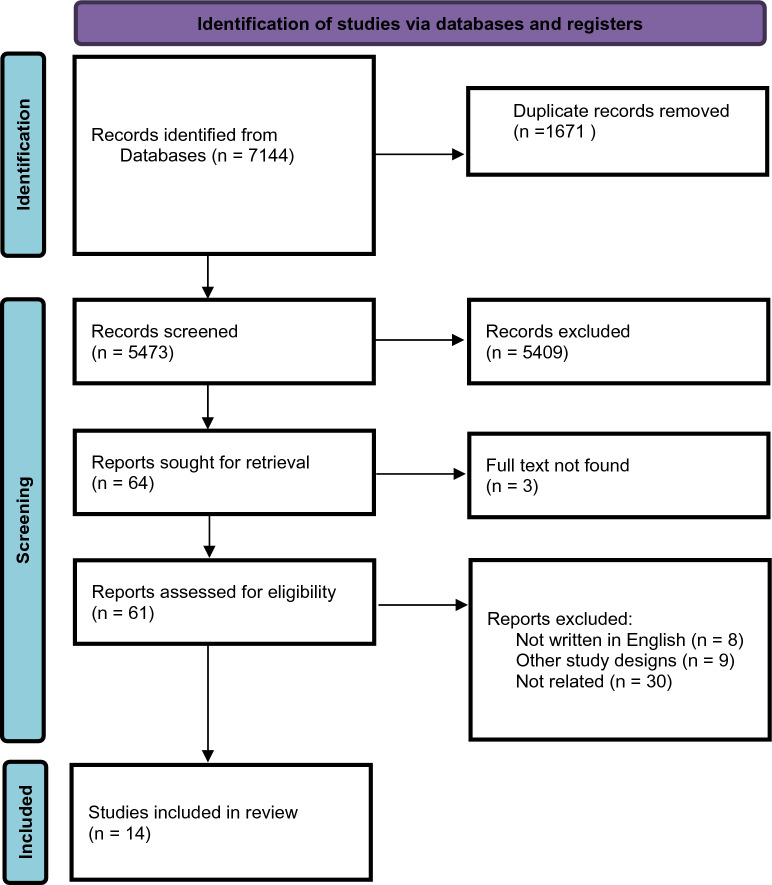
PRISMA 2020 flow diagram for new systematic reviews which included searches of databases and registers only

The initial electronic and manual search rendered 7144 references. After the removal of the duplicates (*n* = 1671) and the irrelevant articles based on their title and abstracts, 64 studies were screened. After full-text screening and implementing inclusion and exclusion criteria, 14 studies were included (Fig. 1). Reviewers (H.B. and R.I.) were in full agreement with screening process.

### Description of studies

Table 1 summarizes the characteristics of included studies in addition to the main findings of each study. From 14 included studies, 8 studies used mammalian xenografts as the intervention group, mostly mammalian xenografts, while 6 studies used fish xenografts. The most reported outcome was re-epithelialization time among all other outcomes. Details of the risk of bias assessment for each study are available in Additional file 1: Tables S2, S3, and S4. One study by Zajicek et al. was considered high-risk and was not included in further meta-analyses.

**Table 1:** Summary of the characteristic findings of the included studies

Author, country, year	Study design	Population	Age	TBSA	Xenograft type	Intervention group	Control group	Main findings
Bovine xenografts								
Tuleubayev et al. [25], Kazakhstan 2022	RCT	68 patients (47 male and 21 female patients)	13.13 ± 5.03% in DBP and 12.11 ± 6.54% in control group	2A Grade burns in both groups	Decellularized bovine peritoneum (DBP)	DBP (31 patients)	Dressings impregnated with 10% Povidone–Iodine (37 patients)	Hospitalization: 10.45 ± 6.15 (DBP) vs. 9.92 ± 6.08 (control) (Not significant) Dressing change: 1.35 ± 0.66 (DBP) vs. 5.22 ± 3 (control) Level of pain: Significantly lower in DBP group Re-epithelialization: 23 of 31 and 24 of 37 patients (not significant)
Porcine xenografts								
Feng et al. [22], China 2006	RCT	40 patients (20 patients in porcine ADM group and 20 in control group)	6 months–88 years	30–94%	Xenogenic ADM (porcine)	Porcine ADM overlapped 2–3 cm at the junctions of separate pieces	Topical antimicrobial agents (povidone–iodine ointment) three times daily and the wound was exposed	Healing time: 9–14 days (porcine) vs. 14–35 days (control) Scar index (3 months): 3.29 ± 1.63 (porcine) vs. 7.75 ± 1.78 (control) Scar index (2 years): 2.77 ± 1.05 (porcine) vs. 7.03 ± 1.24 (control) Dressing changes: No dressing change (porcine)
Zuo et al. [26], China 2016	RCT	6 adult burn patients (4 male and 3 female patients)	24.8 (18–35) years	88.3% ± 5.7% (total burn areas) and 81.6% ± 7.8% (full thickness burn areas)	Porcine xenograft	Fresh pigskins in 2 patients (7/15 operations)	Partial-thickness viable cryopreserved alloskins in 4 patients (8/15 operations)	Autoskin grafted time: 27.3 ± 3.8 days (1^st^ operation), 22.0 ± 5.7 days (2nd operation), and 15.3 ± 1.5 days (3^rd^ operation) Survival percentage: POW 1: 80.0% ± 10.0% (alloskin) vs. 75.7% ± 5.3% (pigskin) (*P* = 0.16) POW 2: 71.2% ± 10.6% (alloskin) vs. 66.4% ± 6.2% (pigskin) (*P* = 0.30) POW 3: 48.7% ± 2.5% (alloskin) vs. 35.0% ± 7.0% (pigskin) (*P* = 0.03) TBSA that survived: 21.8% ± 10.9% (alloskin) vs. 22.4% ± 8.5% (pigskin)
Chen et al. [27], China 2013	RCT	30 patients (20 males and 10 females)	18–60 years	25–60%	Porcine acellular dermal xenograft (ADX)	ADX and split-thickness skin autograft	Split-autologous epidermal skin	Vancouver Scar Scale: Not significant after 1 month but significant after 3, 6, and 12 months Adverse reaction: No ulcer or scar hyperplasia
Zajicek et al. [24], Czech Republic 2011	RCT	86 pediatric patients with superficial scald burns in 2 groups	5 months–7 years	1–35% in total; 10 (6–13) % in Xe-Derma and 7 (4–10) % in Askina THINSite (P = 0.028)	Acellular pig dermis Xe-Derma (porcine)	Xe-Derma (43 patients)	Synthetic hydrogel wound dressing Askina THINSite (43 patients)	Re-epithelialization: 8 (5–10) days (Xe-Derma) vs. 7 (3–10) days (Askina THINSite) (*P* = 0.147) Infection: 6 (Xe-Derma) vs. 10 (Askina THINSite) (*p* = 0.2) Dressing changes: One on day 2 or 3 and outer dressing change every 2 or 3 days
Karlsson et al. [23], Sweden 2022	RCT	24 patients (22 male and 2 female patients)	39 (19–73) years	11 (4–37) % in total; 7 (2–14) % treated with dressings	Porcine xenograft (EZ derm)	EZ derm (11 patients)	Biosynthetic cellulose dressing (BsC) (13 patients)	Healing time: 19 (12–35) in porcine vs. 18 (10–35) in BsC (*P* = 0.7) Hospital stay: 14 (2–28) days in porcine vs. 4 (0–40) days in BsC (*P* = 0.331) Patients’ POSAS total score (12-month follow-up): 45 (31–61) (porcine) vs. 33 (11–55) (BsC) (*P* = 0.39) Observer POSAS total score (12-month follow-up): 20 (13–40) (porcine) vs. 19 (11–25) (BsC) (*p* = 0.45) Infection: 11 of 11 (porcine) vs. 12 of 13 (BsC) (*p* = 1.0)
Hosseini et al. [28], Iran 2008	non-randomized clinical trial	86 burned pediatrics	4 (0.1–15) years	28.8 (10–50) % in conventional and 28.2 (10–54) % in Xenoderm group	Xenoderm: lyophilized pig skin	Xenoderm (51 patients)	Conventional treatments (SSD) (35 patients)	Hospital stay in all patients: 10 (10.8) in Xenoderm vs. 17 (14.4) days (conventional) (*p* = 0.10) Hospital stay in TBSA of 20–39%: 7.5 (3–36) days (Xenoderm) vs. 20 (4–55) days (conventional) (*p* = 0.001) Dressing changes: 6.02 (8.3) (Xenoderm) vs. 12.9 (9.3) times (conventional) (*p* = 0.0005) Mortality: 0 (Xenoderm) vs. 5 (conventional)
Hosseini et al. [29], Iran 2009	non-randomized clinical trial	118 burn patients	26.54 (2–80) in conventional and 26.52 (1–81) years in Xenoderm group	30–75%; 44.7 (31–70) % in conventional and 42.8 (30–72) % in Xenoderm group	Xenoderm: lyophilized pig skin	Xenoderm (65 patients)	Conventional treatment (saline-soaked dressing) (53 patients)	Hospital stay: 18.7 (15.2) (Xenoderm) vs. 24.2 (18.2) days (conventional) (*p* = 0.11) Dressing changes: 10.4 (10.9) (Xenoderm) vs. 18.04 (13.6) (conventional) (*P* = 0.005) Mortality: 7 (10.8%) (Xenoderm) vs. 19 (35.8%) (conventional) (*P* = 0.001)
Fish xenografts								
Li et al. [30], 2021	animal study	30 Sprague–Dawley rats and 5 Bama mini-pigs	6–8-week-old rats	NA (3 round full-thickness skin defects with a diameter of 1.8 cm on each rat and six 5 × 5 cm^2^ square full-thickness skin defect on each pig)	Fish skin-derived ADM (TS-ADM) and porcine ADM (DC-ADM)	TS (alkaline decellularization and γ-irradiation sterilization without freeze-drying)	DC (porcine ADM as active control) and Vaseline gauze (VLGZ as negative control)	Re-epithelialization (2w) in pigs: 23.4% ± 6.3% in TS vs. 10.7% ± 2.6% in VLGZ vs. 12.4% ± 4.6% in DC (significant) Wound dressing: after 2 weeks, TS was significantly easier to remove Wound closure rate (day 35): TS inhibited scar hyperplasia; collagen deposition in group TS was notably higher than other two groups
Lima et al. [31], Brazil 2020	Phase II Pilot RCT	30 pediatrics	2–12 years	< 20%	Tilapia skin (TS)	Tilapia skin (TS)	SSD cream 1%	Re-epithelialization: 10.07 ± 0.46 (TS) vs. 10.47 ± 0.74 days (SSD) Dressing changes: 3.00 ± 0.76 (TS) vs. 9.27 ± 1.39 (SSD) Anesthetic use: lower in TS group
Lima et al. [15], Brazil 2020	Phase II RCT	62 patients (Arm A: SPTB involving < 10% of TBSA; Arm B: SPTB involving 10–20% of TBSA; Arm C: DPTB involving 5–15% of TBSA)	18–50 years	SPTB affecting up to 20% TBSA, or DPTB affecting 5–15% TBSA	Nile Tilapia Fish Skin (NTFS)	NTFS (A: 13, B: 9, and C: 10)	SSD cream 1% group (A: 10, B: 10, and C: 10)	Re-epithelialization: NTFS (A: 9.77 ± 0.83; B: 10.56 ± 1.13; C: 18.10 ± 0.99) and SSD (A: 11.20 ± 0.063; B: 11.70 ± 0.067; C: 21.30 ± 1.42) [significant] Dressing changes: NTFS (A: 2.08 ± 0.28; B: 2.33 ± 0.71; C: 6.10 ± 2.02) and SSD (A: 5.80 ± 0.42; B: 11.00 ± 0.47; C: 20.20 ± 1.69) [significant]
Lima et al. [32], Brazil 2021	Phase III RCT	115 outpatients with SPTB	18–70 years	< 15%	Nile Tilapia Fish Skin (NTFS)	Glycerolized fish skin (NTFS) (57 patients)	SSD cream 1% (58 patients)	Re-epithelialization: 9.7 ± 0.6 in NTFS vs. 10.2 ± 0.9 in SSD (*P* = 0.001) Dressing changes: 1.6 ± 0.7 in NTFS vs. 4.9 ± 0.5 in SSD (*P* < 0.001)
Stone et al. [33], USA 2018	animal study	36 full thickness burn wounds on pigs	NR	NA	omega-3 rich fish skin graft (FSG)	A) FSG (day 0) + 1.5:1 mSTSG (day 7); B) FSG (day 0) + 3:1 mSTSG and FSG applied over the graft (day 7) C) cadaver porcine skin (day 0) + 1.5:1 mSTSG (day 7);		Infection: No infection in FSG Outcome measures, including contraction rates, TEWL measurements, hydration levels, and blood perfusion levels: FSG was similar to cadaver skin Wound healing: The 3:1 mSTSG treated with FSG resulted in similar healing as the wounds treated with the 1.5:1 mSTSG
Stone et al. [34], USA 2021	animal study	6 female Yorkshire pigs (24 deep partial-thickness and 36 full-thickness burns)	NR	NA	Fish skin graft (FSG) or fetal bovine dermis (FBD)	FSG	FBD	Re-epithelialization (day 14): 50.2% in FSG vs. 23.5% in FBD (*P* < 0.005) Reduction in original wound size (day 14): 93.1% in FSG vs. 106.7% in FBD (*P* = 0.005)

ADM, acellular dermal matrix; DPTB, deep partial-thickness burns; mSTSG, meshed split thickness skin grafts; NA, not applicable; NR, not reported; POW, postoperative week; RCT, randomized clinical trial; SPTB, superficial partial-thickness burns; SSD, silver sulfadiazine; TBSA, total body surface area; TEWL, trans-epidermal water loss

## Primary and secondary outcomes

### Mammalian xenografts

#### Re-epithelialization and wound healing

In the Feng et al. trial [22], the time of wound re-epithelialization shortened to 9–14 days in the xenograft group. However, in the patients using topical antimicrobial agents, a scab was reported to form after several days and loosen up in 14–35 days with the healing of the major part of the wound. Karlsson et al. [23] study found no significant difference in the time to more than 95% healing between groups (19 [12–35] days vs. 18 [10–35]; *P* = 0.716). In addition, Zajicek et al. [24] found a non-significant lower time to re-epithelialization in the Xederma group (8 [5–10] days) compared to the control group (7 [3–10] days). In addition, Tuleubayev et al. [25] found a non-significant higher healing time in xenograft (10.45 ± 6.15) compared to control (9.92 ± 6.08) group.

#### Survival of grafts

In Zuo et al. [26] study, the first three tangential excision and skin grafting on subcutaneous tissue wounds (TESGSTW) operations were performed at 2–3, 5–8, and 11–16 day post-injury. The survival percentage of the cryopreserved alloskins and fresh pigskins at the third post operation week were 48.7% ± 2.5% and 35.0% ± 7.0%, respectively, which was significantly different between the two groups.

In the Zajicek et al. study [24], complete conversion from superficial dermal to deep dermal burn wound happened in one child in the Xe-Derma group and in four children in the control group (treated with Askina THINSite, a synthetic hydrogel wound dressing) which was not significantly different. Partial conversion of covered area occurred in 16 patients in the Xe-Derma group and in 18 cases in the Askina THINSite group which the number and extent of converted areas did not have a statistically significant difference.

### Infection

Karlsson et al. [23] reported no significant difference between burn patients using EZderm xenograft and biosynthetic cellulose dressing (BsC) groups. Moreover, in line with this trial, no significant difference between infection rates was reported in Zajicek et al. [24] trial.

#### Scar

In the study conducted by Feng et al. [22], the scar index after 3 months in the porcine acellular dermal matrix (ADM) group was 3.29 ± 1.63 and in the group with povidone–iodine ointment was 7.75 ± 1.78. Moreover, the scar index after 2 years in porcine ADM was 2.77 ± 1.05 and in the povidone–iodine ointment group was 7.03 ± 1.24. In this study, scar hyperplasia was significantly mitigated compared with traditional treatment after a follow-up period of 3 months to 2 years. In the Karlsson et al. [23] study, the median patients’ total POSAS scores for the 12-month follow-up of the scar was 45 (31–61) in the porcine xenograft group and 33 (11–55) in the BsC group; observer’s total POSAS score was 20 (13–40) in the porcine xenograft group and 19 (11–25) in BsC group which none of them were statistically different. In the study by Chen et al. [27], Vancouver scar scale after 1 month was not significantly different between xenograft porcine ADM with split-thickness autograft and the control group treated with split-thickness autograft; however, it was significantly different between the 2 groups after 3, 6, and 12 months.

#### Length of hospital stay

**Fig. 2:**
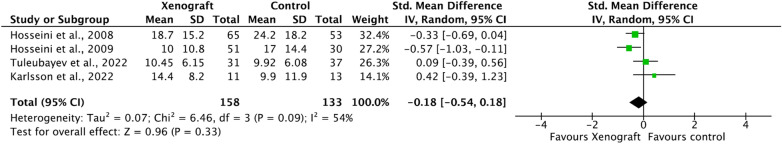
Duration of hospitalization in mammalian xenografts

No significant difference between hospital stays was found in the meta-analysis of 4 studies (SMD [95% CI] = − 0.18 [− 0.54–0.18]; *P* = 0.33; *I*^2^ = 54%; Fig. 2) [23, 25, 28, 29]. Karlsson et al. [23] found no difference between hospital stay between porcine and BsC groups (14 [2–28] days vs. 4 [0–40] days; *P* = 0.331). In Hosseini et al. [28] study in 2008 in pediatric burn patients with TBSA of 20–39%, the median of first admission hospital stay in the conventional and Xenoderm groups were 20 and 7.5 days (*p* = 0.001), respectively. In Hosseini et al. study in 2009 [29], the mean hospital stay was 24.2 days in conventional group compared to 18.7 days in Xenoderm group (*p* = 0.11). In line, Tuleubayev et al. [25] found no significant difference in inpatient days between groups.

#### Number of dressing changes

**Fig. 3:**
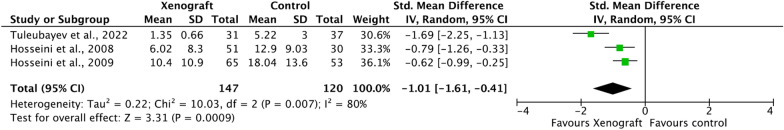
Number of dressing changes in mammalian xenografts

In the meta-analysis of 3 studies [25, 28, 29], we found significantly lower numbers of dressing changes in the xenograft group compared to controls (SMD [95% CI] = − 1.01 [− 1.61–− 0.41]; *P* = 0.0009; *I*^2^ = 80%; Fig. 3). Feng et al. study [22] had no dressing changes in burn patients. In the study conducted by Zajicek et al. [24] dressing change was performed one time on day 2 or 3 and an outer dressing change was done every 2 or 3 days. The number of dressing changes in the trial by Tuleubayev et al. [25] was lower in the xenograft group compared to controls (1.35 ± 0.66 times vs. 5.22 ± 3 times). In Hosseini et al. trial in 2008 [28] in pediatrics, the median number of dressings in the Xenoderm group and conventional group were 6.02 and 12.9 times (*p* = 0.0005), respectively. Finally, in Hosseini et al. study in 2009 [29], the number of dressings was 10.4 in the Xenoderm vs. 18.04 in the conventional group (*P* = 0.0005).

#### Mortality

Two studies by Hosseini et al. compared mortality between the xenograft group and controls; in the Hosseini et al. [28] trial in 2008, 5 deaths happened in the conventional group compared to no death in the Xenoderm group. In line, mortality was higher in the control group in Hosseini et al. [29] trial in 2009 (19 [35%] vs. 7 [10.8%]; *P* = 0.001).

### Fish xenografts

#### Re-epithelialization

**Fig. 4:**
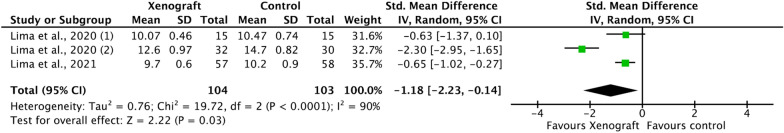
Re-epithelialization in fish xenografts

Day to re-epithelialization was significantly lower in fish xenografts versus controls in the meta-analysis (SMD [95% CI] = − 1.18 [− 2.23–− 0.14]; *P* = 0.03; *I*^2^ = 90%; Fig. 4). In Li et al. study [30], the rate of wound closure between Tilapia skin acellular dermal matrix (TS-ADM) and porcine acellular dermal matrix dressing (DC-ADM) groups was significantly different on day 35 postoperatively. TS-ADM group showed a significant advantage in promoting epithelialization reaching 23.4% ± 6.3% on day 14, while group VLGZ and DC-ADM were only 10.7% ± 2.6% and 12.4% ± 4.6%, respectively. Moreover, TS-ADM enhanced collagen deposition and inhibited scar hyperplasia. In the pilot study by Lima et al. [31], the mean days to complete re-epithelialization was 10.47 ± 0.74 in the SSD group and 10.07 ± 0.46 in the tilapia skin group. Phase II of Lima et al. study [15] was performed in 3 arms, including Arm A with SPTB involving < 10% of TBSA, Arm B with SPTB involving 10–20% of TBSA, and Arm C with DPTB involving 5–15% of TBSA. In their study, re-epithelization days were significantly lower in the Nile Tilapia Fish Skin group (Arm A: 9.77 ± 0.83; Arm B: 10.56 ± 1.13; Arm C: 18.10 ± 0.99) compared to SSD group (Arm A: 11.20 ± 0.063; Arm B: 11.70 ± 0.067; Arm C: 21.30 ± 1.42). In the phase III trial conducted by Lima et al. [32], patients treated with fish skin required fewer days for re-epithelialization (9.7 ± 0.6 days versus 10.2 ± 0.9 days; *p* = 0.001). In the Stone et al. study in 2018 [33], full-thickness burn wounds treated with fish skin graft (FSG) had similar outcome measures (contraction rates, trans-epidermal water loss measurements, hydration levels, and blood perfusion levels) compared to cadaver skin-treated burn wounds. The 3:1 meshed split thickness skin grafts (mSTSG) treated with FSG resulted in similar healing as the wounds treated with the 1.5:1 mSTSG. Stone et al. in 2021 [34] revealed wounds treated with FSGs resulted in faster re-epithelialization beginning at day 10 until day 28; however, this was only significant at day 14 when compared to fetal bovine dermis (FBD) (50.2% vs. 23.5%, *P* < 0.005). The contraction rates were reported as the percentage of original size and a significant reduction in original wound size at day 14 was observed for the FSG when compared to FBD (93.1% vs. 106.7%, *P* < 0.005, respectively).

#### Dressing change

**Fig. 5:**
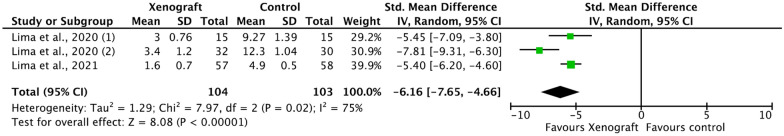
Number of dressing changes in fish xenografts

The number of dressing changes was significantly lower in fish xenografts compared to controls in the meta-analysis (SMD [95% CI] = − 6.16 [− 7.65 – − 4.66]; *P* < 0.001; *I*^2^ = 75%; Fig. 5). In the pilot study by Lima et al. [31], the mean dressing change numbers were 9.27 ± 1.39 in the SSD group and 3.00 ± 0.76 in the tilapia skin group. In the phase II of Lima et al. study [35], the number of dressings changes were significantly lower in the Nile Tilapia fish skin group (Arm A: 2.08 ± 0.28; Arm B: 2.33 ± 0.71; Arm C: 6.10 ± 2.02) compared to SSD group (Arm A: 5.80 ± 0.42; Arm B: 11.00 ± 0.47; Arm C: 20.20 ± 1.69). In the phase III trial conducted by Lima et al. [36], patients treated with fish skin required fewer dressing changes (1.6 ± 0.7 times vs. 4.9 ± 0.5 times; *P* < 0.001).

#### Scar hyperplasia

In the study by Li et al. [30], the results indicated that the use of TS-ADM produced a long-term effect of inhibiting scar hyperplasia. Scar evaluation index can reflect the degree of scar to a certain extent, and scar evaluation index in group TS-ADM was obviously lower than that of group DC-ADM and Vaseline gauze.

#### Infection

In the trial conducted by Stone et al. [33], no infection was detected in wounds treated with FSG.

## Discussion

Compared to other treatments, fish xenografts reduced re-epithelialization time, while meta-analysis was not possible for porcine xenografts. There was no significant difference between mammalian xenograft re-epithelialization times in most studies. In both fish and mammalian xenografts, the number of dressing changes was significantly lower compared to the control groups. Finally, no significant difference in the length of hospital stay in the mammalian xenografts group and controls was found. Although meta-analysis was not possible for other outcomes, most studies reported comparable results in these outcomes.

There are four stages to the normal wound-healing process: hemostasis, inflammation, proliferation, and remodeling [37]. Wound management varies based on the depth and size of the wounds [38]. Wound management can range from simple saline rinses and the use of sterile gauze to complicated surgeries requiring long-term hospitalization. Deep and extensive burns can lead to metabolic disturbances, followed by shock, multi-organ failure, and death. Thus, burns that are deep and/or extensive need intervention and should not be left untreated to prevent further complications [2].

There are three known zones for each burn wound: coagulation, stasis, and hyperemia [39]. The coagulation zone is the area of tissue that is destroyed by a burn. The stasis zone, which surrounds the coagulation zone, has low levels of perfusion and thus can become necrotic and expand in a short period of time after injury. Hence, the process of burn wounds is progressive in both depth and surface and requires intervention. The intricate cellular mechanisms behind burn injury are still not well-known [40]. Microvascular dysfunction is the most known reason for burn wound progression which includes three main mechanisms: (1) vessel thrombosis after vascular damage, (2) inflammatory mediators upregulation, and (3) proapoptotic factors [40]. Although the mechanisms of burn wounds are better known in recent years, there are still many dark spots that increase the importance of experimental studies in finding better treatments for burn patients.

Available treatments for burn wounds include (1) topical silver agents, (2) biological dressings, including amniotic membrane, allografts, xenografts, and bioengineered dressings, (3) enzymatic debridement, and (4) surgery [4]. However, the standard treatment of deep burns is still early excision and using skin grafts [6, 7]. Although autografts showed promising results in treating burns, patients with extensive burns require temporary coverages with allografts, xenografts, and skin substitutes. Thus, evaluating the efficacy of these temporary coverages is essential. Porcine and fish grafts have been reported in the literature as good candidates to be used in burn patients.

In a study by Brown et al. [41] effective pain management and referral to a specialized burn center, were found to be prognostic factors for days to re-epithelialization in addition to known factors, including burn depth, injury mechanism, and TBSA. Moreover, Demling et al. [42] found promising results for silver exposure in reducing days to re-epithelialization. Although conventional treatments are effective in increasing the rate of re-epithelialization, our study found increased or comparable re-epithelialization rates in patients treated with xenografts, which is a promising result and can pave the way for using these grafts as they are more affordable than synthetic grafts.

Burn treatment takes up a large share of financial resources, especially with deep wounds, the treatment is very expensive and imposes a large financial burden on the health system [43]. The number of dressing changes is one of the factors affecting the treatment cost, while lower required dressing changes help the patient by improving the rate of re-epithelization and increasing treatment tolerance in patients [44]. Since our study found a significantly lower number of dressing changes in xenografts, there may be a great benefit in using xenografts in terms of lower cost and improved patient tolerance to treatment.

Length of hospital stay in burn patients is positively correlated with TBSA [45]. Moreover, several models have been proposed for predicting the length of hospital stay and/or defining variables correlated with hospital stay length [46]. According to our results, xenografts do not significantly increase hospital stay compared to other conventional treatments, making them a viable option for patients who have extensive burns.

Our study had some limitations. First, since some studies had not reported sufficient data to perform secondary analysis, meta-analysis was not possible for all outcomes. Second, the control groups in studies were different (e.g., silver sulfadiazine, allograft, or biosynthetic dressings) which can impact the findings of this study. Third, using non-randomized trials can impact the final findings by possible selection bias in individual studies. Finally, different TBSA and other baseline characteristics of patients emphasize the need for designing large clinical trials to better compare xenografts with other conventional treatments.

## Conclusion

In this study, we retrieved that xenografts showed a significantly lower number of dressing changes; the number of days to re-epithelialization showed significant reduction in fish xenografts compared to routine treatment. The beneficial results of xenografts suggest further research in the use of different types of them in burn patients who need a large amount of grafts.

### Research registration unique identifying number (UIN)

1. Name of the registry: PROSPERO.

2. Unique Identifying number or registration ID: CRD42022373748.

3. Hyperlink to your specific registration (must be publicly accessible and will be checked): https://www.crd.york.ac.uk/prospero/display_record.php?ID=CRD42022373748

### Supplementary Information

**Additional file 1: Table S1.** Search strategy and keywords. **Table S2.** Quality assessment of randomized clinical trials using cochrane tool of risk of bias assessment (RoB). **Table S3.** Quality assessment of non-randomized clinical trials using risk of bias in non-randomized studies – of interventions (ROBINS-I). **Table S4.** Quality assessment of non-randomized clinical trials using SYRCLE.

## Data Availability

The data sets used and analyzed during the current study that are not presented in the manuscript nor Additional files, are available from the corresponding author on reasonable request.
