# Supplementary material for: The clinical outcomes of xenografts in the treatment of burn patients: a systematic review and meta-analysis
Source: Eur J Med Res. 2023 Nov 16;28:524. doi: 10.1186/s40001-023-01505-9 (PMC10652578; doi:10.1186/s40001-023-01505-9)
Supplement: Supplementary file 1 — Additional file 1: Table S1. Search strategy and keywords. Table S2. Quality assessment of randomized clinical trials using cochrane tool of risk of bias assessment (RoB). Table S3. Quality assessment of non-randomized clinical trials using risk of bias in non-randomized studies – of interventions (ROBINS-I). Table S4. Quality assessment of non-randomized clinical trials using SYRCLE. [file 40001_2023_1505_MOESM1_ESM.docx]

**Additional File 1**

**Table S1. Search Strategy and Keywords**

| Database | Query | Number of hits |
| --- | --- | --- |
| PubMed | (((((((((xeno*[Title/Abstract]) OR (heterograft[Title/Abstract])) OR ("animal transplant"[Title/Abstract])) OR ("EZ derm"[Title/Abstract])) OR (porcine[Title/Abstract])) OR (pig[Title/Abstract])) OR (fish[Title/Abstract])) OR (bovine[Title/Abstract])) OR (ovine[Title/Abstract])) AND ((burn*[Title/Abstract]) OR ("burn injury"[Title/Abstract])) | 2077 |
| Cochrane Library | (xeno*):ti,ab,kw OR (heterograft):ti,ab,kw OR ("animal transplant"):ti,ab,kw OR ("EZ derm"):ti,ab,kw OR (porcine):ti,ab,kw OR (pig):ti,ab,kw OR (fish):ti,ab,kw OR (bovine):ti,ab,kw OR (ovine):ti,ab,kw ) AND ( (burn*):ti,ab,kw OR ("burn injury"):ti,ab,kw ) | 145 trials and 6 reviews |
| Scopus | (TITLE-ABS-KEY(xeno*) OR TITLE-ABS-KEY(heterograft) OR TITLE-ABS-KEY("animal transplant") OR TITLE-ABS-KEY("EZ derm") OR TITLE-ABS-KEY(porcine) OR TITLE-ABS-KEY(pig) OR TITLE-ABS-KEY(fish) OR TITLE-ABS-KEY(bovine) OR TITLE-ABS-KEY(ovine)) AND (TITLE-ABS-KEY(burn*) OR TITLE-ABS-KEY("burn injury")) | 1403 |
| Web of Science | ((((((((TS=(xeno*)) OR TS=(heterograft)) OR TS=("animal transplant")) OR TS=("EZ derm")) OR TS=(porcine)) OR TS=(pig)) OR TS=(fish)) OR TS=(bovine)) OR TS=(ovine) AND (TS=(burn*)) OR TS=("burn injury") | 3513 |
| Total hits |  | **7144** |

**Table S2. Quality Assessment of Randomized Clinical Trials Using Cochrane Tool of Risk of Bias Assessment (RoB)**

| Study | Randomization process | Deviations from intended interventions | Missing outcome data | Measurement of the outcome | Selection of the reported result | Overall Bias |
| --- | --- | --- | --- | --- | --- | --- |
| Feng et al. (1) |  |  |  |  |  |  |
| Zuo et al. (2) |  |  |  |  |  |  |
| Chen et al. (3) | 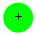 | 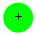 | 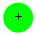 | 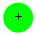 | 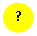 | 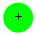 |
| Zajicek et al. (4) |  | \|  \| \| --- \| | \| 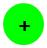 \| \| --- \| | \| 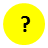 \| \| --- \| | \| 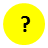 \| \| --- \| | \|  \| \| --- \| |
| Karlsson et al. (5) |  |  |  |  |  |  |
| Tuleubayev et al. (6) |  |  |  |  |  |  |
| Lima et al. (7) |  |  |  |  |  |  |
| Lima et al. (8) |  |  |  |  |  |  |
| Lima et al. (9) |  |  |  |  |  |  |

**Table S3. Quality Assessment Of Non-randomized Clinical Trials Using Risk Of Bias In Non-randomized Studies – of Interventions (ROBINS-I)**

| **Authors** | **Bias due to confounding** | **Bias in selection of participants into the study** | **Bias in classification of interventions** | **Bias due to deviations from intended interventions** | **Bias due to missing data** | **Bias in measurement of outcomes** | **Bias in selection of the reported result** | **Overall Bias** |
| --- | --- | --- | --- | --- | --- | --- | --- | --- |
| **Hosseini, 2008 (10)** | Low | Low | Low | NI | Low | Moderate | Low | Low |
| **Hosseini, 2009 (11)** | Low | Low | Low | NI | Low | Moderate | Low | Low |

**Table S4. Quality Assessment of Non-randomized Clinical Trials Using SYRCLE**

| Risk of bias domains | Stone  (12) | Stone (13) | Li (14) |
| --- | --- | --- | --- |
| 1) Was the allocation sequence adequately generated and applied? | Yes | Yes | Yes |
| 2) Were the groups similar at baseline or were they adjusted for confounders in the analysis? |  |  |  |
| *Was the distribution of relevant baseline characteristics balanced for the intervention and control groups? | Yes | Yes | Yes |
| *If relevant, did the investigators adequately adjust for unequal distribution of some relevant baseline characteristics in the analysis? | Yes | Unclear | Yes |
| *Was the timing of disease induction adequate? | Yes | Yes | Yes |
| 3) Was the allocation to the different groups adequately concealed during? | Yes | Unclear | Unclear |
| 4) Were the animals randomly housed during the experiment? |  |  |  |
| *Did the authors randomly place the cages or animals within the animal room/facility? | Unclear | Unclear | Unclear |
| *Is it unlikely that the outcome or the outcome measurement was influenced by not randomly housing the animals? | Unclear | Unclear | Unclear |
| 5) Were the caregivers and/or investigators blinded from knowledge which intervention each animal received during the experiment? | Yes | Unclear | Unclear |
| 6) Were animals selected at random for outcome assessment? | Yes | Unclear | Yes |
| 7) Was the outcome assessor blinded? |  |  |  |
| *Was blinding of the outcome assessor ensured, and was it unlikely that blinding could have been broken? | Yes | Unclear | Unclear |
| *Was the outcome assessor not blinded, but do review authors judge that the outcome is not likely to be influenced by lack of blinding? | - | - | Yes |
| 8) Were incomplete outcome data adequately addressed? |  |  |  |
| *Were all animals included in the analysis? | Unclear | Yes | Yes |
| *Were the reasons for missing outcome data unlikely to be related to true outcome? (e.g., technical failure) | Unclear | - | - |
| *Are missing outcome data balanced in numbers across intervention groups, with similar reasons for missing data across groups? | Unclear | - | - |
| *Are missing outcome data imputed using appropriate methods? | Unclear | - | - |
| 9) Are reports of the study free of selective outcome reporting? |  |  |  |
| *Was the study protocol available and were all of the study’s pre-specified primary and secondary outcomes reported in the current manuscript? | Yes | No | Yes |
| *Was the study protocol not available, but was it clear that the published report included all expected outcomes (i.e. comparing methods and results section)? | - | Yes | - |
| 10) Was the study apparently free of other problems that could result in high risk of bias? |  |  |  |
| *Was the study free of contamination (pooling drugs)? | Yes | Yes | Yes |
| *Was the study free of inappropriate influence of funders? | Yes | Yes | Yes |
| *Was the study free of unit of analysis errors? | Yes | Unclear | Yes |
| *Were design-specific risks of bias absent? | Yes | Unclear | Unclear |
| *Were new animals added to the control and experimental groups to replace drop-outs from the original population? | Unclear | No | Unclear |

**References**

1. Feng XS, Tan JJ, Pan YG, Wu QH, Ruan SB, Shen R, et al. Control of hypertrophic scar from inception by using xenogenic (porcine) acellular dermal matrix (ADM) to cover deep second degree burn. Burns. 2006;32(3):293-8.

2. Zuo HB, Song GD, Shi W, Jia J, Zhang YH. Observation of viable alloskin vs xenoskin grafted onto subcutaneous tissue wounds after tangential excision in massive burns. Burns & Trauma. 2016;4:10.

3. Chen X, Feng X, Xie J, Ruan S, Lin Y, Lin Z, et al. Application of acellular dermal xenografts in full-thickness skin burns. Exp Ther Med. 2013;6(1):194-8.

4. Zajicek R, Matouskova E, Broz L, Kubok R, Waldauf P, Königova R. New biological temporary skin cover Xe-Derma(®) in the treatment of superficial scald burns in children. Burns. 2011;37(2):333-7.

5. Karlsson M, Elmasry M, Steinvall I, Huss F, Olofsson P, Elawa S, et al. Biosynthetic cellulose compared to porcine xenograft in the treatment of partial-thickness burns: A randomised clinical trial. Burns. 2022;48(5):1236-45.

6. Tuleubayev B, Ogay V, Anapiya B, Zhylkibayev A, Saginova D, Koshanova A, et al. Therapeutic Treatment of 2A Grade Burns with Decellularized Bovine Peritoneum as a Xenograft: Multicenter Randomized Clinical Trial. Medicina (Kaunas). 2022;58(6).

7. Lima Júnior EM, Moraes Filho MO, Forte AJ, Costa BA, Fechine FV, Alves A, et al. Pediatric Burn Treatment Using Tilapia Skin as a Xenograft for Superficial Partial-Thickness Wounds: A Pilot Study. J Burn Care Res. 2020;41(2):241-7.

8. Lima EM, de Moraes MO, Costa BA, Rohleder AVP, Rocha MBS, Fechine FV, et al. Innovative Burn Treatment Using Tilapia Skin as a Xenograft: A Phase II Randomized Controlled Trial. J Burn Care Res. 2020;41(3):585-92.

9. Lima EM, de Moraes MO, Costa BA, Fechine FV, Vale ML, Diogenes AKD, et al. Nile Tilapia Fish Skin-Based Wound Dressing Improves Pain and Treatment-Related Costs of Superficial Partial-Thickness Burns: A Phase III Randomized Controlled Trial. Plast Reconstr Surg. 2021;147(5):1189-98.

10. Hosseini SN, Mousavinasab SN, Rahmanpour H, Shoghli A. Xenoderm versus 'conventional' treatment in pediatrics burns. Int J Pharmacol. 2008;4(1):46-50.

11. Hosseini SN, Mousavinasab SN, Rahmanpour H, Fallahnezhad M. A biological dressing versus 'conventional' treatment in patients with massive burns: a clinical trial. Ulus Travma Acil Cerrahi Derg. 2009;15(2):135-40.

12. Stone R, Larson D, Wall J, Florell K, Dillon H, Magnusson S, et al. IMPROVED HEALING OF DEEP PARTIAL THICKNESS BURN WOUNDS WITH OMEGA-3 RICH FISH SKIN DERMIS COMPARED TO FETAL BOVINE DERMIS. Wound Repair Regen. 2018;26(4):A6-A.

13. Stone R, Larson D, Wall J, Florell K, Dillon H, Magnusson S, et al. Omega-3 Rich Fish Skin Grafts Reduce Donor Skin Requirements for Full Thickness Burns. Wound Repair Regen. 2018;26(1):A22-A3.

14. Li DS, Sun WQ, Wang T, Gao YL, Wu JL, Xie ZP, et al. Evaluation of a novel tilapia-skin acellular dermis matrix rationally processed for enhanced wound healing. Mater Sci Eng C-Mater Biol Appl. 2021;127:16.
